# Intra- and inter-rater reliability of Fugl-Meyer Assessment of Lower Extremity early after stroke

**DOI:** 10.1016/j.bjpt.2020.12.002

**Published:** 2020-12-17

**Authors:** Edgar D. Hernández, Sandra M. Forero, Claudia P. Galeano, Nubia E. Barbosa, Katharina S. Sunnerhagen, Margit Alt Murphy

**Affiliations:** a Departamento del Movimiento Corporal Humano, Universidad Nacional de Colombia, Bogota, Colombia; b Central Military Hospital of Colombia, Bogota, Colombia; c Institute of Neuroscience and Physiology, Clinical Neuroscience, Rehabilitation Medicine, Sahlgrenska Academy, University of Gothenburg, Gothenburg, Sweden

**Keywords:** Item-level reliability, Leg motor activity, Scale, Stroke rehabilitation, Svensson’s method

## Abstract

**Background:**

The Fugl-Meyer Assessment of Lower Extremity (FMA-LE) is a widely used and recommended scale for evaluation of post-stroke motor impairment. However, the reliability of the scale has only been established by using parametric statistical methods, which ignores the ordinal properties of the scale.

**Objective:**

To determined intra- and inter-rater reliability of the FMA-LE at item and summed score level early after stroke.

**Methods:**

Sixty patients (mean age 65.9 years, median FMA-LE 29 points) admitted to the hospital due to stroke were included. The FMA-LE was simultaneously, but independently, scored by three experienced and trained physical therapists randomly assigned into pairs, on two consecutive days, between 4 to 9 days post stroke. A rank-based statistical method for paired ordinal data was used to assess the level of agreement and systematic and random disagreements.

**Results:**

The item-level reliability was high (percentage of agreement [PA] ≥75%). Two items (ankle dorsiflexion during flexor synergy and normal reflex activity) showed some systematic disagreement in intrarater analysis. A satisfactory intrarater reliability (PA ≥70%) was reached for all summed scores when a 1- or 2-point difference was accepted between ratings.

**Conclusion:**

The FMA-LE is a reliable tool for assessment of motor impairment both within and between raters early after stroke. The scale can be recommended not only for use in Spanish speaking countries, but also internationally. A unified international use of FMA-LE would allow comparison of stroke recovery outcomes worldwide and thereby potentially improve the quality of stroke rehabilitation.

## Highlights

• The Spanish FMA-LE can be recommended for evaluation of motor impairment in stroke.
• Intra- and interrater reliability of the Spanish FMA-LE was excellent.
• Wider use of FMA-LE would allow worldwide comparisons of stroke recovery.

## Introduction

Stroke is the leading cause of disability worldwide.^1^ The incidence and global burden of stroke is increasing, particularly in low- and middle-income countries.^2^ Motor impairment is the most prominent impairment after stroke as it affects planning, production, and execution of movements in the contralateral arm and leg.^3^ Muscle weakness, altered muscle coupling, and co-activation are common motor deficits that often can be more prominent in distal parts of the body particularly when corticospinal descending and ascending neural pathways are involved in the injury.^4^ Motor impairments in lower extremity influence walking ability, walking speed, as well as static and dynamic postural control.^5^, ^6^, ^7^, ^8^, ^9^, ^10^ The Copenhagen unselected cohort study in stroke showed that approximately 65% of individuals had leg paresis at admission and that 55% had remaining paresis at discharge from an inpatient rehabilitation unit.^11^ Similarly, about 63% and 36% were either not able to walk or needed assistance at admission and discharge, respectively.^11^ These numbers indicate that assessment of lower extremity paresis is essential in all stages of stroke to fully understand the mechanisms of motor control and its consequences on walking ability, postural control, and activities of daily living.

The Fugl-Meyer Assessment of Lower Extremity (FMA-LE) is a widely used scale for assessment of motor function after stroke.^12^, ^13^ The scale is recognized as a gold standard and is recommended both for clinical use and research worldwide.^14^, ^15^ The scale includes assessment of reflex activity, voluntary movements within and outside of synergies, ability to perform isolated movement, and coordination. The FMA-LE measures a unidimensional underlying construct, motor impairment, and poses hierarchical properties.^16^, ^17^ This means that the scale is valid for determining level of motor function in people with stroke.

Reliability, cross-sectional and predictive validity, as well as responsiveness of the FMA-LE have been demonstrated by several previous studies.^15^, ^18^ Excellent intra- and inter-rater reliability of the FMA-LE in the sub-acute phase (intraclass correlation coefficient [ICC], 0.95−0.99) and chronic phase (ICC 0.88–0.95) has been reported.^18^, ^19^, ^20^ The scoring of each item of the FMA-LE is done at the ordinal level (0–2) and the total score is calculated as a sum-score. The summing of ordinal scores does not result in a number that is valid for making quantitative analysis on reliability or any other comparisons.^21^ Therefore, the results from studies using parametric statistics on ordinal scales, such as ICC, should be interpreted with caution. Furthermore, the reliability of the FMA-LE needs to be established by using methods suited for ordinal data to verify the agreement and not only association between different raters. For predictive purposes the use of single items or sub-scores of longer scales has become of great interest among researchers and clinicians.^22^, ^23^, ^24^, ^25^, ^26^ Thus, there is a need to establish the intra- and inter-rater reliability at all levels.

The FMA-LE was recently translated into Colombian Spanish following the protocol and manual according to the original English/Swedish version.^27^ Because the psychometric properties of a scale are dependent on the language, population, and setting, there is a need to assess reliability and validity of the Spanish version of the FMA-LE. Thus, the aim of this study was to evaluate the intra- and inter-rater reliability of the FMA-LE at the item and summed score level in people early after stroke.

## Methods

### Participants

This study, investigating intra- and inter-rater reliability, involved a sample of 60 patients consecutively admitted to the Central Military Hospital of Colombia in Bogota during a 17-month period due to stroke. Inclusion criteria were: first event stroke, upper or lower extremity hemiparesis, admitted to the hospital between 4 to 9 days post stroke, age between 18 and 90 years. Exclusion criteria were: other disorders such as blindness, deafness, amputation of lower or upper limb, cerebellar stroke, not able to cooperate in FMA testing due to impaired cognition or other severe medical condition. The severity of the stroke at hospital admission was assessed by the National Institutes of Health Stroke Scale (NIHSS)^28^ and the disability level at discharge by the Modified Rankin Scale.^29^

The study protocol was endorsed by the Research Ethics Committee of the Central Military Hospital, Bogota, Colombia (Act No. 9, 12 June 2013) and a signed informed consent was obtained from all participants or their family member. The data collection was conducted between November 2014 and April 2016. The STROBE (Strengthening the Reporting of Observational studies in Epidemiology) guidelines^30^ and the checklist for reliability evaluation from the consensus-based standards for selection of health status measurement instruments (COSMIN) were followed to ensure the methodological quality of the study.^31^ The statistical rank invariant method used in the current study to determine reliability is not listed in the COSMIN, but it is a valid alternative for determination of reliability in ordinal paired data.^32^, ^33^, ^34^ The sample size estimation was based on previous studies using the same statistical methodology.^35^, ^36^

### Fugl-Meyer Assessment of Lower Extremity

The FMA-LE assesses lower extremity motor function including reflex activity, movement within and outside synergy patterns, and speed/coordination.^13^ It comprises 17 items in two subscales: Lower Extremity (E) and Speed/Coordination (F), which are scored on a 3-level ordinal scale (0 points: none; 1 point: partial; 2 points: full). The item scores are then summed. The maximum score for the Lower Extremity Subscale is 28 points and for Speed/Coordination 6 points. The total summed score of 34 points indicates normal function. The protocol used for FMA-LE assessment is available at www.neurophys.gu.se/rehabmed.

Three physical therapists were randomly assigned into pairs of two to perform the assessments. All raters had more than 20 years of clinical experience and underwent training on the FMA-LE prior to the start of the study. All raters were involved in the translation process of the FMA from English into Spanish, which also included joint practical training with guidance of experts and data collection for a previous pilot study.^27^ The patient’s performance on the FMA-LE was simultaneously, but independently, scored by one pair of raters on two consecutive days. The first assessment was performed between 4 to 9 days post stroke. During the first assessment one of the raters was acting as test leader (i.e. instructing the patient and scoring) and the other as observer (scoring by observing). These roles were switched on the second assessment day. The examiners did not communicate during the testing session or afterwards regarding the scoring. The scoring protocols were stored in sealed envelopes until the data collection was completed.

### Statistical analysis

Descriptive statistics were calculated for the background data. Floor and ceiling effects for the FMA-LE were defined as more than 15% of patients receiving the lowest or highest score on the scale.^18^

For the intra- and inter-rater reliability, a rank invariant method specially designed for analysis of systematic and non-systematic disagreements in paired ordinal data was used^32^, ^33^, ^34^ (the software is available at http://avdic.se/svenssonsmetod.html). This method was preferred over the weighted kappa, because the latter fails to identify the systematic disagreements and ignores the rank invariant properties of ordinal data.^37^, ^38^ The weighted kappa also assumes that the raters have equal skill level, which means that systematic disagreements are ignored.^37^, ^38^ In addition, the weighted kappa value depends on the choice of weights and is sensitive to the number of categories, which means that the value increases when the number of categories decreases.^38^

The degree of agreement was determined by using the percentage of agreement (PA) in which agreement ≥70% was considered satisfactory.^39^ For the summed scores, a minimum disagreement in points to reach at least 70% PA was also calculated. The systematic disagreement between raters was expressed as relative position (RP), the relative concentration (RC), and the relative rank variation (RV).^32^ The RP indicates the extent to which the distribution of scores from an assessment is systematically shifted towards higher or lower categories. The RC shows whether the scores are more or less concentrated towards the central categories of the scale compared to the other assessment. The RP and RC values can vary from −1 to 1, where 0 means no difference between raters. Values within −0.1 and 0.1 were considered negligibly small with reference to clinical relevance, while values outside this range were considered as clinically relevant disagreements.^38^ The RV indicates disagreement caused by individual variability and varies between 0 and 1 and a value <0.1 means that the difference is negligible. Statistically significant disagreement of RP, RC, and RV was indicated with a 95% confidence interval (95% CI) that did not include the value zero. The statistical software also produced Receiver Operating Characteristic (ROC) curve for each comparison which were used to visually evaluate the detected systematic disagreements. Concave or convex curves indicated disagreement in position and S-shaped curve that raters concentrated their assessment differently on the scale categories. The reliability was considered to be excellent when all systematic and non-systematic disagreements were statistically non-significant within the limits stated above.

## Results

Out of 105 eligible patients, 45 were excluded due to: limited ability to follow test instructions (n = 21), cerebellar stroke (n = 8), severe multi-impairment (n = 6), discharged (n = 5), prior stroke (n = 4), deceased (n = 1). All 60 patients (31 men and 29 women, mean age of 65.9 years) included in the study were able to perform the FMA-LE (Table 1). The majority (93%) had ischemic stroke and 7% had haemorrhagic stroke. The FMA-LE scores of the study group ranged from 4 to 34 points. The FMA-LE showed no floor or ceiling effect (9 patients received full score of 34 points).

**Table 1:** Demographic and clinical characteristics (n = 60).

Characteristics	Value
Age, years, mean ± SD	65.9 ± 17.3
Sex, male/female, n (%)	31/29 (52%/48%)
Ischemic/hemorrhagic stroke, n (%)	55/5 (93%/7%)
Right/left hemiparesis, n (%)	33/27 (55%/45%)
Thrombolysis, n	8
Hospitalization days, mean ± SD	12 ± 10
Days post stroke to first assessment, mean ± SD	5.95 ± 2.73
Modified Rankin Scale, median (Q1–Q3)	2 (1–4)
0–2 Mild or non-significant disability, n	35
3–5 Moderate to severe disability, n	25
NIHSS Scale, median (Q1–Q3)	5 (3−10)
Mild 0–5, n	25
Moderate to severe 6–24, n	22
Patients without NIHSS scorings, n	13

Discharged from hospital	
Home, n	56
Homecare, n	1
Intermediate care, n	1
Died in hospital, n	2

Fugl Meyer Assessment of Lower Extremity (FMA-LE)	
FMA-LE, 1st occasion, median (Q1–Q3)	29 (26–31)
FMA- LE, 2nd occasion, median (Q1–Q3)	29.5 (27–31)

*Abbreviations*: NIHSS, National Institutes of Health Stroke Scale.

### Intrarater reliability

The intrarater reliability was calculated separately for all three raters. At the item level the PA across all raters was above 75% for all tested items (Table 2). The rank invariant analysis of agreement revealed statistically significant disagreement of RP (≥0.1) for the ankle dorsiflexion within synergies in supine position (E.II) and for test of normal reflex activity (E.V) in one of the raters (Table 3). The ankle dorsiflexion in supine (E.II) and in standing position (E.IV) showed also a tendency towards non-negligible disagreements (asymmetric 95% CI) in concentration and position, respectively. All these disagreements were positive, which indicates that a higher category was systematically more frequently used at the second occasion for these items sub scores or total scores. No individual disagreement measured as random variance was noted across raters.

**Table 2:** Percentage of agreement (PA%) within each rater (A, B, and C) and between test occasions.

E. Lower extremity	Intrarater agreement (PA %)			Interrater agreement (PA %)	
	Rater A	Rater B	Rater C	Test occasion 1	Test occasion 2
	n = 40	n = 38	n = 38	n = 60	n = 60
I. Reflex activity					
Flexors	97	97	97	100	100
Extensors	100	100	97	100	100

II. Within synergies, supine					
Hip flexion	95	94	92	95	97
Knee flexion	97	100	97	100	100
Ankle dorsiflexion	95	88	**89**^a^	92	95
Hip extension	92	97	97	100	98
Hip adduction	97	100	94	98	98
Knee extension	97	100	100	97	97
Ankle plantar flexion	95	88	89	98	93
*SUM E II, range 0−14 points*	90	79	83	88	90

III. Mixed synergies, sitting					
Knee flexion	95	97	92	98	95
Ankle dorsiflexion	90	91	86	93	95
*SUM E III, range 0–4 points*	90	91	83	92	93

IV. Little or no synergies, standing					
Knee flexion to 90°	92	82	81	95	93
Ankle dorsiflexion	77	76	86	88	95
*SUM E IV, range 0−4 points*	**69**^a^	68	**72**^a^	85	92
*SUM E IV, 1-point difference*	100	95	94	–	–

V. Normal reflex activity					
Knee flexors, patellar, achilles	92	91	**83**^a^	100	100
SUM E, range 0−28 points	62	62	**53**^a^	78	85
SUM E, 1-point difference accepted	87	79	69	–	–
SUM E, 2-point difference accepted	94	86	82	–	–

F. Coordination/speed					
Tremor	87	100	97	93	92
Dysmetria	85	91	83	90	92
Time	77	76	75	95	97
*SUM F, range 0−6 points*	62	68	64	83	87
*SUM F, 1-point difference accepted*	90	97	91	–	–

TOTAL E–F, range 0–34 points	51	47	**50**^a^	75	80
TOTAL E–F, 1-point difference accepted	77	76	69	–	–
TOTAL E–F, 2-point difference accepted	92	82	80	–	–

*Abbreviations*: PA, percentage of agreement, RP, relative position.

a Statistically significant disagreement where the absolute value of RP is ≥0.1 and the 95% confidence interval does not include 0 are marked in bold.

**Table 3:** The rank invariant analysis of intrarater agreement within raters A, B, and C.

E. Lower extremity	Rater A		Rater B		Rater C	
	RP (95% CI)	RC (95% CI)	RP (95% CI)	RC (95% CI)	RP (95% CI)	RC (95% CI)
I. Reflex activity						
Flexors	0.03 (−0.02, 0.07)	–	0.03 (−0.03, 0.09)	–	0.03 (−0.03, 0.08)	–
Extensors	0	–	0	–	0.03 (−0.03, 0.08)	–
SUM E I (0–4 points)	0.03 (−0.02, 0.07)	–	0.03 (−0.03, 0.09)	–	0.03 (−0.03, 0.08)	–

II. Within synergies, supine position						
Hip flexion	0.05 (−0.02, 0.11)	−0.04 (−0.09, 0.02)	0 (−0.08, 0.08)	0 (−0.03, 0.03)	0.03 (−0.06, 0.11)	−0.01 (−0.07, 0.04)
Knee flexion	0.02 (−0.02, 0.07)	−0.02 (−0.06, 0.02)	0	0	0.03 (−0.02, 0.08)	−0.02 (−0.07, 0.02)
Ankle dorsal flexion	0.04 (−0.02, 0.10)	−0.03 (−0.08, 0.02)	0.04 (−0.04, 0.11)	−0.10 (−0.21, 0.01)^b^	**0.10** **(0.01, 0.12)**^a^	−0.06 (−0.14, 0.02)
Hip extension	0 (−0.07, 0.06)	−0.03 (−0.12, 0.06)	0 (−0.01, 0)	−0.03 (−0.09, 0.03)	0.03 (−0.02, 0.08)	−0.02 (−0.07, 0.02)
Hip adduction	0.02 (−0.02, 0.07)	−0.03 (−0.08, 0.03)	0	0	0.05 (−0.02, 0.12)	−0.04 (−0.11, 0.03)
Knee extension	0 (−0.01, 0)	−0.03 (−0.08, 0.03)	0	0	0	0
Ankle plantar flexion	0.04 (−0.02, 0.10)	−0.03 (−0.08, 0.02)	0.02 (−0.07, 0.11)	−0.05 (−0.14, 0.04)	0.05 (−0.05, 0.15)	−0.03 (−0.09, 0.03)
SUM E II (0−14 points)	0.04 (−0.01, 0.10)	0	0.06 (−0.05, 0.18)	0	0.09 (0, 0.19)	0

III. Mixed synergies, sitting position						
Knee flexion	0 (−0.06, 0.06)	0 (−0.05, 0.05)	−0.03 (−0.09, 0.03)	−0.02 (−0.07, 0.03)	0 (−0.07, 0.07)	0 (−0.08, 0.08)
Ankle dorsiflexion	0 (−0.09, 0.09)	0 (−0.06, 0.06)	−0.01 (−0.08, 0.07)	−0.03 (−0.11, 0.04)	−0.01 (−0.10, 0.09)	−0.03 (−0.12, 0.06)
SUM E III (0−4 points)	0 (−0.09, 0.09)	0 (−0.06, 0.06)	−0.01 (−0.08, 0.06)	−0.05 (−0.16, 0.07)	0 (−0.10, 0.10)	0 (−0.10, 0.09)

IV. Little or no synergy, standing position						
Knee flexion to 90°	0.06 (−0.01, 0.13)	−0.06 (−0.13, 0.01)	0.05 (−0.07, 0.17)	−0.04 (−0.14, 0.07)	0.07 (−0.04, 0.19)	−0.05 (−0.16, 0.07)
Ankle dorsiflexion	0.11 (−0.01, 0.23)^b^	−0.09 (−0.21, 0.03)	−0.05 (−0.19, 0.10)	−0.06 (−0.17, 0.05)	0.07 (−0.01, 0.16)	−0.05 (−0.17, 0.07)
SUM E IV (range 0−4 points)	**0.12** **(0.02, 0.23)**^a^	−0.05 (−0.16, 0.06)	0.01 (−0.11, 0.13)	−0.06 (−0.19, 0.07)	**0.11** **(0.01, 0.21)**^a^	−0.05 (−0.21, 0.11)

V. Normal reflex activity						
Knee flexors, patellar, Achilles	0.03 (−0.06, 0.11)	0	0.03 (−0.07, 0.13)	0	**0.17** **(0.05, 0.29)**^a^	0
SUM E (0–28 points)	0.06 (0, 0.12)	0	0.07 (−0.05, 0.19)	0	**0.13** **(0.03, 0.23)**^a^	0

F. Coordination/speed						
Tremor	−0.09 (−0.18, 0)	0.09 (−0.02, 0.19)	0	0	−0.03 (−0.08, 0.02)	0.02 (−0.02, 0.05)
Dysmetria	0.04 (−0.06, 0.13)	0.01 (−0.08, 0.11)	0.07 (−0.01, 0.15)	0.02 (−0.06, 0.10)	0.09 (−0.02, 0.20)	0.01 (−0.08, 0.09)
Time	0 (−0.11, 0.10)	0.05 (−0.10, 0.21)	0.09 (−0.04, 0.22)	0.07 (−0.07, 0.21)	−0.06 (−0.20, 0.09)	0 (−0.12, 0.13)
SUM F (0–6 points)	−0.03 (−0.10, 0.05)	0.04 (−0.06, 0.15)	0.09 (−0.01, 0.19)	0.11 (−0.03, 0.25)^b^	0.01 (−0.10, 0.11)	0 (−0.13, 0.14)
Total E–F (0–34 points)	0.05 (−0.01, 0.11)	0	0.10 (−0.02, 0.22)^b^	0	**0.11** **(0.02, 0.21)**^a^	0

*Abbreviations*: PA, percentage of agreement, RP, relative position; RC, relative concentration; CI, confidence interval.

Absolute values of RP/RC ≤ 0.01 are assigned value 0.

a Statistically significant disagreement (absolute value of RP/RC ≥ 0.1 and 95%CI does not include 0, marked in bold).

b Tendency towards a non-negligible disagreement (absolute value of RP/RC ≥ 0.1 and asymmetric 95%CI around 0).

At the summed score level (Table 2), 79%–100% agreement was reached for movements performed within and mixed synergies (E.II and E.III), and 62%–72% was reached for movements performed with little or no synergy (E.IV) and coordination/speed (F). A disagreement in relative position was revealed for the sum-score of little or no synergy (E.IV) (Table 3). For the summed score E including all motor items (possible maximum score of 28 points), the agreement within raters varied between 53% and 62% (Table 2). When all items were summed to a total score E–F (maximum score of 34 points), the agreement varied between 47% and 51%. The lower PA values in the summed scores were expected because the number of possible categories is larger. However, 69%–87% PA was reached for sum-score E, and for total sum-score E–F when a 1-point difference between test occasions was accepted. Thus, a satisfactory intrarater reliability at sum-score levels was reached when 1- or 2-points difference between test-occasions was accepted.

### Interrater reliability

The FMA-LE scores for each item showed high level of agreement (all above 88%) between raters at both test occasions (Table 2). The PA for summed scores of each section varied between 83% and 100%. PA for the summed score E was between 78% and 85%, and for the total sum-score E–F between 75% and 80%. Disagreements were negligible or not statistically significant (Table 4). No individual disagreements measured as random variance was noted across raters.

**Table 4:** The rank invariant analysis of interrater agreement between test occasions.

E. lower extremity	Test occasion 1		Test occasion 2	
	RP (95% CI)	RC (95% CI)	RP (95% CI)	RC (95% CI)
I. Reflex activity				
Flexors, extensors	0	0	0	–

II. Within synergies, supine position				
Hip flexion	−0.05 (−0.10, 0)	0.02 (−0.01, 0.06)	0.03 (−0.01, 0.07)	−0.02 (−0.04, 0.01)
Knee flexion	0	0	0	0
Ankle dorsal flexion	−0.04 (−0.10, 0.02)	0 (−0.06, 0.05)	−0.03 (−0.06, 0.02)	0.04 (−0.01, 0.09)
Hip extension	0	0	0.02 (−0.01, 0.04)	−0.02 (−0.05, 0.01)
Hip adduction	0.02 (−0.01, 0.04)	−0.01 (−0.04, 0.01)	0.02 (−0.01, 0.04)	−0.02 (−0.05, 0.01)
Knee extension	−0.01 (−0.04, 0.02)	0.03 (−0.01, 0.07)	0 (−0.04, 0.04)	0 (−0.03, 0.03)
Ankle plantar flexion	−0.02 (−0.04, 0.01)	0.01 (−0.01, 0.03)	−0.01 (−0.06, 0.04)	0.03 (−0.02, 0.08)
SUM E II, 0−14points	−0.06 (−0.11, −0.01)^a^	0	−0.01 (−0.05, 0.04)	0

III Mixed synergies, sitting position				
Knee flexion	−0.02 (−0.05, 0.01)	0.01 (−0.01, 0.03)	−0.02 (−0.07, 0.04)	0.01 (−0.03, 0.05)
Ankle dorsiflexion	−0.05 (−0.10, 0)	0.01 (−0.05, 0.06)	0.03 (−0.02, 0.07)	−0.04 (−0.09, 0.01)
SUM E III, 0–4 points	−0.05 (−0.09, 0)	0.01 (−0.05, 0.07)	0.03 (−0.02, 0.07)	−0.04 (−0.09, 0)

IV. Little or no synergy, standing position				
Knee flexion to 90°	0 (−0.06, 0.07)	−0.03 (−0.05, 0.01)	0.03 (−0.03, 0.08)	0.01 (−0.04, 0.05)
Ankle dorsiflexion	−0.01 (−0.01, 0.06)	−0.02 (−0.08, 0.04)	−0.01 (−0.06, 0.03)	0.01 (−0.03, 0.05)
SUM E IV, 0–4 points	0 (−0.07, 0.07)	−0.06 (−0.12, 0.01)	0 (−0.04, 0.05)	−0.01 (−0.05, 0.03)

V. Normal reflex activity				
Knee flexors, patellar, Achilles	0	0	0	0
SUM E, range 0−28 points	−0.03 (0.08, 0.01)^a^	0	0.02 (−0.02, 0.05)	0

F. coordination/speed				
Tremor	−0.02 (−0.08, 0.04)	−0.01 (−0.06, 0.04)	0.02 (−0.05, 0.09)	0.03 (−0.01, 0.07)
Dysmetria	−0.01 (−0.06, 0.05)	0.03 (−0.04, 0.10)	0 (−0.06, 0.06)	0.05 (−0.01, 0.11)
Time	0.02 (−0.03, 0.07)	0.01 (−0.04, 0.05)	0 (−0.04, 0.04)	0 (−0.04, 0.04)
SUM F, 0−6 points	0 (−0.05, 0.05)	0.03 (−0.04, 0.11)	−0.01 (−0.05, 0.04)	0.02 (−0.05, 0.09)
Total E–F, range 0–34 points	−0.03 (−0.07, 0.01)	0	0 (−0.03, 0.04)	0

*Abbreviations*: PA, percentage of agreement, RP, relative position, RC, relative concentration.

Absolute values of RP/RC ≤ 0.01 are signed value 0.

a Statistically significant but negligible disagreement (absolute RP/RC < 0.1 and 95%CI does not include 0).

## Discussion

This study demonstrated that the FMA-LE is a reliable clinical instrument for evaluation of motor function after stroke. Apart from two items in which systematic disagreements were observed, the item level intra- and inter-rater reliability was excellent. The interrater reliability at the summed score levels was excellent, although, in the intrarater analysis, a shift towards higher scores at the second test occasion was observed in few cases. The level of agreement was satisfactory for the summed sub-scores and the total score when 1- or 2-point difference between ratings was accepted.

The results of this study confirm the excellent intra- and inter-rater reliability, at item and summed score level, previously shown for the Fugl-Meyer Assessment of Upper Extremity (FMA-UE).^36^ Furthermore, the item level reliability of FMA-LE was even higher compared to upper extremity assessment. In FMA-LE, only two items, the ankle dorsiflexion during flexor synergy and normal reflex activity, demonstrated statistically significant systematic disagreement within-raters when assessed one day apart early after stroke. Similar to the reliability study of the FMA-UE,^36^ a systematic shift towards higher scores at the second test occasion was observed, which is indicative for possible spontaneous recovery at this early stage of stroke. To improve reliability of items and the sum scores that showed systematic disagreements, clearer guideline and training might be needed.

The intra- and inter-rater reliability of the FMA-LE has shown to be excellent using parametric statistical analysis.^18^, ^19^, ^20^ In a study with similar sample size to ours, an ICC score of 0.95 was reported for the FMA-LE in a chronic stroke population.^18^ Intrarater reliability ICC was as high as 0.99 among expert raters and 0.91 when experts were compared to trained raters.^19^ Equally high ICC was reported for four physical therapist who underwent joint training for the FMA-LE.^20^ The results from our study extend these findings further by showing that when the scale was analysed as an ordinal scale, the interrater reliability at the total score level was excellent (PA 80% and no observed disagreements). The intrarater reliability of the total FMA-LE score demonstrated some systematic shift towards higher scores at the second test occasion. Correspondingly, only 50% of ratings had the exact same score at both occasions. Our results showed, however, that when a 1-point difference of the total score was accepted, a 69% agreement was reached. Likewise, a 2-point difference resulted in an 80% agreement between ratings. These absolute values on the expected variance of the summed scores between ratings can be useful for clinicians when making distinction between real improvement and random measurement errors in repeated assessments. The absolute disagreement in the FMA-LE scores was clearly under the reported minimal important difference,^18^, ^19^ which confirms the stability of this scale. It is important to note that the results from this study are primarily applicable for the hospitalized patients in the acute and subacute phase of stroke.^40^

The item-level reliability has previously only been determined for the FMA-UE,^36^ in which an agreement between 79% to 100% was found between and within raters. These results are analogous to the found agreement for the FMA-LE in this study. The item-level reliability, in particular of the motor items of FMA-LE, was also high in a small sample of patients with subacute stroke included in a transcultural validation of FMA scale into Italian language.^41^ The item-level reliability is important to establish together with the reliability of the summed scores. The use of single items of the FMA-UE scale has been of great interest in prediction of motor recovery post stroke.^22^, ^24^ Our results demonstrate that most of the single items of FMA-LE can reliably be used in repeated measures. This opens an opportunity to evaluate the potential of single items or combination of a set of items as potential indices for prediction of motor or functional outcome poststroke.

### Strengths and limitations

The strength of this study is the large sample size and the consecutive inclusion of a representative cohort of patients admitted to acute hospital care and rehabilitation after a first-time stroke. The initial motor impairment, assessed 4–9 days post stroke onset, showed that most of the patients had moderate to mild lower extremity impairment, with the FMA-LE scores covering the entire range of the scale without showing floor or ceiling effects. The characteristics of the study sample, however, needs to be considered when generalizing the results.

The COSMIN checklist recommends the use of weighted kappa for analysis of reliability in ordinal data. In the current study, a rank invariant method especially designed for paired ordinal data was used. This choice was based on the fact that, different from weighted kappa, the rank invariant method can identify systematic disagreement and considers the invariant properties of ordinal data. Weighted kappa assumes that the raters have equal skill level, which means that systematic disagreements are ignored.^37^, ^38^ Additionally, the weighted kappa value is sensitive to the choice of weights and the number of categories, meaning that a higher value can be achieved when the number of categories is low.^38^ Thus, the rank-based method used in the current study have some advantages compared to the weighted kappa statistics.

The relatively short time interval, 1 day, between the first and second assessment used for intrarater reliability, might have caused a recall bias for the raters. This was, however, considered to have lesser influence than a possible improvement of motor function at this early stage of stroke. Indeed, the results suggest that even a one day interval might have been too long for establishing intrarater reliability early after stroke, since a positive systematic shift was observed. This shift could also be caused by the learning effect, occurring both in patients and raters when getting familiar with testing procedures at the second occasion. To minimize the bias in scorings, prior training is needed. In this study, all three physical therapists conducting the assessments had undergone joint training prior to data collection. Additionally, each rater had extensive clinical experience with stroke rehabilitation. Training together with clear protocols and instructions, preferably in the form of an instructional video, are needed to ensure high level reliability.

## Conclusions

The FMA-LE showed excellent intra- and inter-rater reliability in a representative cohort of patients early after stroke in the inpatient rehabilitation setting. The FMA-LE can be recommended as a reliable tool for assessment of motor impairment both at item- as well as summed score levels. A wider use of the FMA-LE both in Spanish speaking countries and worldwide in the inpatient care settings would strengthen the reporting of stroke outcomes and make comparisons between regions and countries possible, thereby improving the quality of care.

## Conflicts of interest

The author declares no conflicts of interest.
